# Association of intercellular adhesion molecule 1 (ICAM1) with diabetes and diabetic nephropathy

**DOI:** 10.3389/fendo.2012.00179

**Published:** 2013-01-22

**Authors:** Harvest F. Gu, Jun Ma, Karolin T. Gu, Kerstin Brismar

**Affiliations:** ^1^M1:03 Rolf Luft Center for Diabetes and Endocrinology Research, Department of Molecular Medicine and Surgery, Karolinska Institutet, Karolinska University HospitalStockholm, Sweden; ^2^Department of Anesthesiology, Anzhen Hospital, Capital Medical UniversityBeijing, People’s Republic of China; ^3^Viktor Rydberg Gymnasium Odenplan SchoolStockholm, Sweden

**Keywords:** intercellular adhesion molecule 1, diabetic nephropathy, end-stage renal disease, type 1 diabetes mellitus, type 2 diabetes mellitus

## Abstract

Diabetes and diabetic nephropathy are complex diseases affected by genetic and environmental factors. Identification of the susceptibility genes and investigation of their roles may provide useful information for better understanding of the pathogenesis and for developing novel therapeutic approaches. Intercellular adhesion molecule 1 (ICAM1) is a cell surface glycoprotein expressed on endothelial cells and leukocytes in the immune system. The *ICAM1* gene is located on chromosome 19p13 within the linkage region of diabetes. In the recent years, accumulating reports have implicated that genetic polymorphisms in the *ICAM1* gene are associated with diabetes and diabetic nephropathy. Serum ICAM1 levels in diabetes patients and the *icam1* gene expression in kidney tissues of diabetic animals are increased compared to the controls. Therefore, ICAM1 may play a role in the development of diabetes and diabetic nephropathy. In this review, we present genomic structure, variation, and regulation of the *ICAM1* gene, summarized genetic and biological studies of this gene in diabetes and diabetic nephropathy and discussed about the potential application using ICAM1 as a biomarker and target for prediction and treatment of diabetes and diabetic nephropathy.

## INTRODUCTION

Diabetes mellitus is a group of metabolic diseases in which the patients have high blood glucose levels. Its epidemic has become a national and global crisis. Based upon the figures today, at least 366 million people at the worldwide have diabetes. By the year 2030, this number is expected to be double (Bonow and Gheorghiade, 2004; Wild et al., 2004; Cornell and Dorsey, 2012; Lam and LeRoith, 2012). There are two major types of diabetes. Type 1 diabetes (T1D), previously called juvenile diabetes or insulin-dependent diabetes, develops on the basis of autoimmune destruction of pancreatic β-cells, which results in insulin deficiency. It mostly affects young people (<20 years old) but occurs also in adults (Lightfoot et al., 2012). Type 2 diabetes (T2D) is the most common form of diabetes and accounts for approximately 85–90% of all diabetic patients. In T2D, hyperglycemia results from a combination of impaired insulin secretion and insulin resistance. When the pancreatic β-cells loose the ability to compensate for insulin resistance in liver, skeletal muscle, and adipose tissues, hyperglycemia becomes manifest (Alberti and Zimmet, 1998). Diabetes patients often develop macro- and/or micro-vascular complications. Diabetic nephropathy (DN) is one of serious complications and occurs in 30–40% of diabetic patients (Heerspink and de Zeeuw, 2011; Marshall, 2012). This diabetic complication is characterized by pathophysiological changes in glomerular hyperfiltration, renal hypertrophy, tubular function and then progress to proteinuria and reduction of glomerular filtration rate (GFR). The patients with DN exhibit persistent proteinuria, hypertension, declining renal function, and increased premature mortality largely as a result of cardiovascular disease. DN is the most common single cause of end-stage renal disease (ESRD). Once overt DN occurs, it progresses slowly or rapidly to the most advanced stage of chronic kidney disease which needs dialysis or transplantation treatment (Marshall, 2004; Shields and Maxwell, 2010; Weil et al., 2010; Thomas and Groop, 2011). The treatment cost for diabetes patients has been increasing staggering in the recent decades and becomes a further burden of the healthcare system. Diabetes and DN are multi-factorial diseases, which are influenced by both genetic and environmental factors (Satko et al., 2005; Pitkäniemi et al., 2007; Ashcroft and Rorsman, 2012; Gonzalez-Bulnes and Ovilo, 2012; Morahan, 2012). Therefore, identification of the susceptibility genes in development of diabetes and diabetic complications and investigation of their roles are of importance to provide useful information for improvement of the prevention and medication programs.

Intercellular adhesion molecule 1 (ICAM1, OMIM: 147840) is a cell surface glycoprotein and expressed in endothelial cells and leukocytes in the immune system. This endothelial- and leukocyte-associated transmembrane protein has been known for its importance in stabilizing cell–cell interactions and facilitating leukocyte endothelial transmigration. Recently, the accumulating reports from genetic studies in diabetic patients with and without DN and from biological studies with diabetic animal models have implicated that ICAM1 may play a role in the pathogenesis of diabetes and DN. In this review, we will summarize the genetic and pathophysiological relevance of ICAM1 and discuss about the possible role of ICAM1 in the development of diabetes and DN as well as the perspectives of the ICAM1 research.

## GENOMIC DNA STRUCTURE, mRNA AND PROTEIN OF ICAM1

**FIGURE 1:**
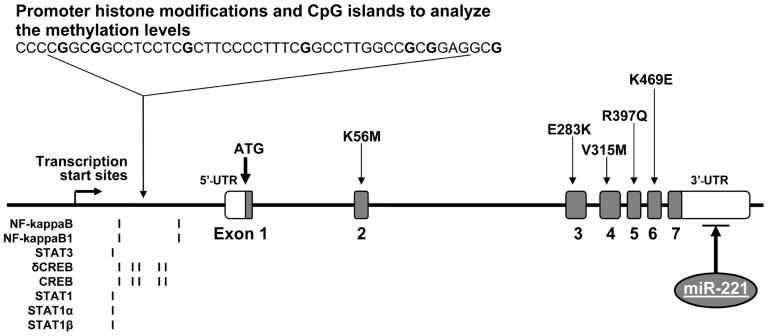
**Genomic DNA structure, variation, and regulation of the *ICAM1* gene**. The *ICAM1* gene is consisted of seven exons and six introns. There are several non-synonymous SNPs, which reside one each in exons 2–6. In the gene promoter, there are CpG islands for methylation and epigenetically silence by histone modifications as well as transcription binding sites for the proteins, including NF-kappaB, NF-kappaB1, STAT3, δCREB, STAT1, STAT1α, and STAT1β. In addition, microRNA (miR-221) in the region of 3′-UTR may down-regulate the *ICAM1* gene expression.

The *ICAM1* gene (GeneID: 3383) is located in chromosome 19p13.2 and spans 15,775 base pairs (bp) along the short arm of this chromosome (10,381,517–10,397,291 bp from *pter*). Its aliases include cluster of differentiation 54 (CD54) and cell surface glycoprotein P3.58 (BB2). **Figure 1** demonstrates genomic structure, regulation, and variation of the *ICAM1* gene. There are seven exons and six introns in the *ICAM1* gene. A relatively large un-translation region (UTRs) resides respectively at both 5′ and 3′-sequences of the gene, while the translation start point is located in exon 1. From the 2–6 exon, there is one non-synonymous single-nucleotide polymorphism (SNP) each, by which the amino acid changes of ICAM1 protein are caused. ICAM1 is a transmembrance glycoprotein molecule of the immunoglobulin superfamily and characterized by five distinct immunoglobulin-like domains, a transmembrance domain and a cytoplasmic tail (van de Stolpe and van der Saag, 1996). ICAM1 protein is 505 amino acids in the length, the molecule weights between 80 and 114 kDa depending upon the levels of glycosylation, which varies among cell types and environments (Newman et al., 1990). Interestingly, there are several transcription binding proteins including NF-kappaB, NF-kappaB1, STAT3, δCREB, STAT1, STAT1α, and STAT1β, which may up-regulate the *ICAM1* gene activity (Roebuck and Finnegan, 1999). MicroRNA (miR-221) may down-regulate the gene expression in the region of 3′-UTR of *ICAM1* (Gong et al., 2011). There are CpG islands for methylation in the promoter of the *ICAM1* gene. A study in tumor endothelial cells has demonstrated that the *ICAM1* gene activity can be epigenetically silenced by promoter histone modifications (Hellebrekers et al., 2006). All regulatory factors are of importance to control the ICAM1 activities in immune-related processes. Our research has been focused on investigating whether alterations in the *ICAM1* gene structure and function are associated with the development of diabetes and DN.

## LINKAGE OF THE *ICAM1* GENE TO DIABETES AND DIABETIC NEPHROPATHY

Diabetes and DN are multi-factorial diseases, which are influenced by both genetic and environmental factors. To search for the susceptibility genes for the diseases, genome-wide scan linkage analysis has been used. This is a family-based approach to investigate if the genetic markers such as microsatellites or SNPs that span the whole genome and co-segregate with disease phenotypes. Microsatellites are simple repeats of 1–6 bp in genome and (CA)n is the most common form. A total of ~30,000 microsatellites present high levels of inter- and intra-specific polymorphisms and distribute in whole genome (often in intergenic DNA regions and rarely in the sequences of the genes). SNPs are the substitutions of nucleotides in genomic DNA. In general speaking, bi-allelic SNPs are the most common type. Tri-allelic ones and small insertions/deletions are also included. C/T is the most common SNP in the human genome. SNPs reside in the coding region of the genes are called cSNPs, which include non-synonymous SNPs (with amino acid changes) and synonymous SNPs (without amino acid changes). Most of SNPs are located in non-coding regions of the genes, including promoter, intron, and UTRs. SNPs in the promoter region may alter transcription binding site and thereby affect the transcriptional activity of the gene. Moreover, a number of SNPs are found in inter-genic sequences. Up to date, more than 40 million SNPs are recorded in the public SNP databases, which are freely available for research use. Using the genetic markers, the linkage analysis allows us to identify the genome that is transmitted within families along with the disease phenotypes of interest at a genome-wide scale. Based on finding a statistical signal, the probability of co-segregation of a disease with a chromosomal locus is given (Gulcher, 2012). In this approach of genome-wide scan, highly polymorphic genetic markers distributed across the genome are genotyped in large family pedigrees or in affected and discordant sibling pairs.

By using the approach of genome-wide scan and linkage analyses, several chromosomal regions including 19p13 have been predicted to link with diabetes and DN. Mein et al. (1998) have previously conducted genome wide scan analysis in 93 affected sibpair families and 263 multiplex families from the UK and indicated that loci in chromosome 19p13 are linked to T1DM. Later on, the linkage of T1DM to chromosome 19p13 is replicated by the study with 2658 affected sib-pairs in USA (Concannon et al., 2009). Interestingly, lipid-related traits such total cholesterol, triglycerides and low-density lipoprotein (LDL) concentrations in T2DM are linked to chromosome 19p13 and this finding has been replicated in Caucasians, African-American and Hispanic families (Imperatore et al., 2000; Adeyemo et al., 2005; Malhotra and Wolford, 2005). Several interesting candidate genes, including insulin receptor, resistin and *ICAM1*, are involved in the region of chromosome 19p13. The linkage of LDL concentrations to chromosome 19p13 has been replicated in the study with 612 individuals from 28 Amish families in USA (Pollin et al., 2004). Furthermore, Leon et al. (2007) have performed a genome-wide scan study in 1251 African Americans (AA) and 1129 European Americans (EA) hypertensive siblings from the Hypertension Genetic Epidemiology Network study and indicated that loci in chromosome 19p are linked with albumin to creatinine ratio (ACR) when both AA and EA subjects are combined in the analyses. Particularly, Kathiresan et al. (2008) have analyzed the genome-wide scan data from three studies including 8816 T2D subjects and found six new loci associated with LDL, cholesterol, HDL, and triglycerides. One of the loci is located in chromosome 19p13. Previously, Arya et al. (2006) have conducted a genome-wide scan and linkage study and suggested that loci in both chromosomal arms of 19p13.2 and 19q13.4 may be linked to birth weight in T2D families of both Mexican Americans and EA. By the analyses of the combined metabolic syndrome and echocardiographic factors, Kraja et al. (2008) have found the linkage of blood pressure in T2D patients of AA and EA with the region chromosome 19p13. Taking together with the information from above briefly described genome-wide scan studies, it is clear that there are the loci in chromosome 19p may confer the susceptibility risk to diabetes and DN.

## ASSOCIATION OF THE *ICAM1* GENETIC POLYMORPHISMS WITH DIABETES AND DIABETIC NEPHROPATHY

Several research groups including ours have reported the genetic association studies of the *ICAM1* gene in T1DM and DN. Guja et al. (1999) reported that the transmission of the G allele of SNP K469E (A/G) is increased in Romanian T1DM families. One year later, Nishimura et al. (2000) replicated that this K469E polymorphism is associated with adult-onset T1DM in a Japanese population. However, the association of K469E polymorphism in the *ICAM1* gene with T1DM was not found in Danish, Finnish, and British Caucasian (Nejentsev et al., 2000). Furthermore, Nejentsev et al. (2003) demonstrated that another synonymous SNP G241R in the *ICAM1* gene was associated with T1DM. All these previous studies were designed for analysis of one or two SNP(s). In order to ascertain whether the *ICAM1* genetic polymorphisms are associated with T1DM and DN, we conducted the comprehensive genetic association studies. The studied SNPs including K469E and G241R were selected based upon the information of their position in the *ICAM1* gene and their linkage disequilibrium (LD) values in the HapMap. Our data from single marker association analyses indicated that K469E and another intronic polymorphism (rs281432) were significantly associated with T1DM in Swedish Caucasians (Ma et al., 2006). Interestingly, these two SNPs are located in intron 2 and exon 6, respectively. According to the data of pair-wise LD values for SNPs in the *ICAM1* gene, a relatively strong LD (/D′/ ≥ 0.7) existed to extend over the region between these two SNPs. Due to the large 5′- and 3′- UTRs in exons 1 and 7, the LD block covers almost the whole coding region of the gene. Further multiplex marker association analysis was done and the common haplotype C-A constructed by C allele from K469E and A allele from rs281432 was found to be associated with T1D (Ma et al., 2006). Later on, we found that K469E polymorphism in the *ICAM1* is associated with DN in T1D patients of Americans of European descent and selected from the Genetic of Kidney Diseases in Diabetes (GoKinD) study (Mueller et al., 2006). However, no association of G241R in the *ICAM1* gene with T1DM and DN in Swedish and GoKinD populations was found (Ma et al., 2008). In patients with T2D, the K469E polymorphism in the *ICAM1* gene was found to associate with plasma fibrinogen levels and diabetic retinopathy (Kamiuchi et al., 2002; Yokoyama et al., 2005; Liu et al., 2006; Petrovic et al., 2008; Vinita et al., 2012). Up to date, there is, however, no report regarding the association of *ICAM1* genetic polymorphism with DN in T2D.

**FIGURE 2:**
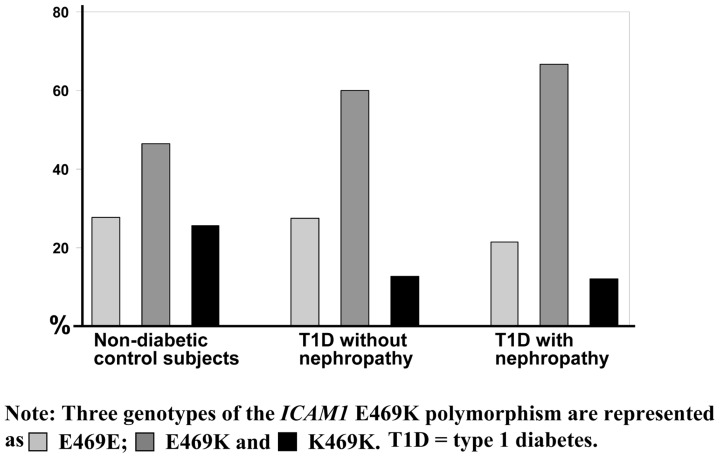
**Genotype distribution of the *ICAM1* K469E polymorphism**. The genotype distribution of the* ICAM1* K469E polymorphism is represented from a genetic association study in Swedish population (Ma et al., 2006). Three genotypes of the *ICAM1* K469E polymorphism are shown in as light gray color for K469K, gray for K469E, and dark for E469E. Obviously, the heterozygous index is high compared to the percentage of homozygous and increased from the group of non-diabetic control subjects, to type 1 diabetes (T1D) patients without diabetic nephropathy and the patients with diabetic nephropathy.

Interestingly, we have observed that genotype distribution of K469E polymorphism in the *ICAM1* gene presents a high heterozygous index (~50%; Ma et al., 2006). **Figure 2** represents the genotype distribution of the *ICAM1* E469K polymorphism in Swedish non-diabetic control subjects, T1D patients without and with DN. From non-diabetic control subjects, to T1D patients without DN and the patients with DN, the frequencies of the carriers with heterozygous genotype are increased, while the carriers with 469E homozygous genotype decreased. In order to avoid the possibility that high heterozygous index may be caused by genotyping errors, we confirmed the genotyping experiments with two different techniques such as dynamic allele-specific hybridization (DASH) and pyrosequencing (Ronaghi et al., 1998; Howell et al., 1999). In the human genome, there are segmental duplications (duplicons) with >90% sequence similarity between the copies, which may cause specific allelic and genotypic diversities, such as high heterozygous index in complex diseases (Venter et al., 2001; Shaw and Lupski, 2004). To ascertain whether K469E SNP is involved in a duplicon, we further performed a cloning and sequencing analysis and found that no duplication resides in the gene region (Ma et al., 2006). K469E is a non-synonymous SNP in exon 6 of the *ICAM1* gene, which causes the amino acid changes of the ICAM1 protein. We have submitted ICAM1 amino acid sequences with K469 and 469E alleles respectively into SWISS-MODEL (Peitsch, 1995; Arnold et al., 2006) to understand the changes of ICAM1 protein. There are 532 amino acids in the protein sequence of ICAM1, K469 is wild-type and has 100% identified homology. Compared to the DIMER image of wild ICAM1 protein, however, the structure of ICAM with mutant 469E is significantly changed. Although the modeling analysis implicates that the K469E polymorphism in the *ICAM1* gene may have functional effect, further investigation with transfection of 469E allele into cells such human embryonic kidney (HEK) 293A or with *icam1* knock-out mouse model is necessary in order to further understand the pathogenic mechanism.

## POSSIBLE ROLE OF ICAM1 IN DEVELOPMENT OF DIABETES AND DIABETIC NEPHROPATHY

In general speaking, ICAM1 proteins act as ligands and the primary receptors for ICAM1 are integrins, which mediate cell–cell interactions and allow signal transduction. Specifically, ICAM1, unlike most integrin-binding proteins, does not contain an RGD (Arg-Gly-Asp) motif to promote integrin binding (van de Stolpe and van der Saag, 1996), but is targeted to two integrins of the β2 subunit family, i.e., leukocyte adhesion protein-1 (LFA-1) and Mac-1 (integrin, alpha M; Janeway, 2001). Thus, based upon the interaction with these two molecules, ICAM1 has a role for two important immune-related functions: T lymphocytes activation and leukocyte–endothelial cell interaction. The role of ICAM1 in the development of diabetes and DN has not been fully explored. Recent studies, however, have provided the information to predict that ICAM1 is involved in the pathogenesis of diabetes and DN (Sahakyan et al., 2010a,b).

**FIGURE 3:**
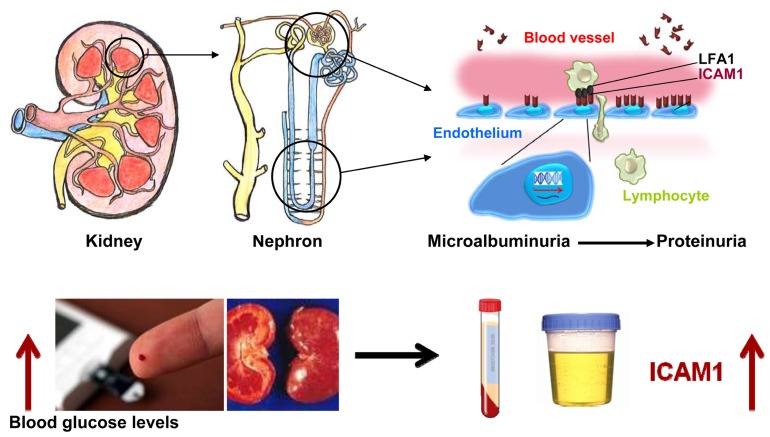
**Possible role of ICAM1 in the development of diabetic nephropathy**. Under a diabetic condition with hyperglycemia, the *ICAM1* gene transcription in the nuclei is increased and the *ICAM1* gene expression on the surface of endothelium cells is up-regulated. ICAM1 protein binding activity with leukocyte adhesion protein-1 (LFA-1) is increased and more lymphocytes from blood are transferred into cells of glomeruli and peritubular capillaries of nephron in kidney. Consequently, serum ICAM1 levels are increased. Injury in kidney glomeruli and tubular is occurred and the proteins are released to urine.

Diabetic nephropathy is a progress disease, which is categorized into stages based upon urinary albumin excretion (UAE) values. The early phase, which can be reversed, is microalbuminuria. The reduction of renal function begins with proteinuria. Clinical investigation has demonstrated that soluble ICAM1 levels in stored blood samples from T1D patients are higher compared to non-diabetic control subjects. High ICAM1 levels in T1D patients are associated with a relative risk of 1.67 (95 CI 0.96–2.92, *P* = 0.03) of developing incident sustained microalbuminuria after adjustment for baseline age, sex, duration of diabetes, and randomized treatment assignment (Lin et al., 2008). Furthermore, Astrup et al. (2008) have reported that soluble ICAM1 levels are associated with all-caused mortality and cardiovascular morbidity in T1D patients with DN. The similar findings have been observed in T2D patients. Soluble ICAM1 levels are significantly correlated with albuminuria in T2D patients (Rubio-Guerra et al., 2007). T2D patients with diabetic micro-angiopathic complications have higher soluble ICAM1 levels in comparison with diabetic group without micro-angiopathic complications and healthy control subjects (Mastej and Adamiec, 2008). The findings from clinical investigations have been supported by studies with diabetic animal models such as the db/db mice and streptozotocin-induced rats. Compared to non-diabetic rats, serum and urinary ICAM1 levels in streptozotocin-induced rats are found to be increased, which are parallel with the elevation of UAE (Qian et al., 2008). Furthermore, evidence has indicated that ICAM1 is overexpressed in glomeruli diabetic rats (Watanabe et al., 2011) and in tubular epithelial cells of kidney in T2D db/db mice (Kosugi et al., 2009). Therefore, ICAM1 may play a role in the development of diabetes DN and possible mechanism is shown in **Figure 3**. In a diabetic condition with hyperglycemia, the *ICAM1* gene transcription in the nuclei is increased and the *ICAM1* gene expression on the surface of endothelium cells is up-regulated. ICAM1 binding activity with LFA-1 is increased and more lymphocytes from blood are transferred into cells in glomeruli and peritubular capillaries of nephron in kidney. Consequently, injure of kidney glomeruli and tubular occurs and the proteins are excreted to urine. In this figure, however, two questions still remain. First, how is ICAM1 gene activity stimulated by high blood glucose levels? Second, how does ICAM1 elevation cause kidney tubular and glomeruli injury?

### ICAM1 AS A BIOMARKERS FOR PREDICTION OF DIABETIC NEPHROPATHY

Biomarkers are substances and structures that can be measured in biological samples such as urine, blood, saliva, DNA, and protein. A biomarker can be used as an indicator of a particular disease state such as diabetes and DN (Caveney and Cohen, 2011). Currently, the biomarker used for prediction and diagnosis of DN is UAE. This biomarker, however, is less valuable for early prediction and diagnosis of DN. Therefore, researchers have searched for novel biomarkers of DN in order to improve the diagnosis approach and prevention program (Hellemons et al., 2012). As we described above, genetic and biological studies have implicated that ICAM1 plays a role in the development of diabetes and DN. First, the *ICAM1* gene is located in a linkage region with diabetes and DN. Second, the K469E polymorphism in the *ICAM1* gene is associated with diabetes and DN. Third, serum ICAM1 levels are gradually increased from low levels in normal albuminuria to high levels in micro-albuminuria and to even higher levels in proteinuria. Therefore, we concluded that ICAM1 is associated with diabetes and DN. This molecule is most likely a useful biomarker for prediction of endothelial dysfunction in diabetes and DN. Further evaluation of ICAM1 as a biomarker in a large cohort of T1D and T2D patients with and without DN needs to be done.

### ICAM1 AS A TARGET FOR DRUG DEVELOPMENT

ICAM1 is a molecule involved in many pathways including anti-inflammation. Although the picture of ICAM1 involvement and interaction is complex, experiments have indicated that inhibition of the ICAM1 gene expression may improve the progress of diabetes and DN. Glucagon-like peptide-1 (GLP-1) has various extra-pancreatic actions, in addition to its enhancement of insulin secretion from pancreatic islets. Kodera et al. (2011) have demonstrated that GLP-1 receptor agonist, exendin-4, decreases the ICAM1 gene expression and ameliorates albuminuria, glomerular hyperfiltration, glomerular hypertrophy, and mesangial matrix expansion in the diabetic rats without changing blood pressure or body weight. Furthermore, Matsui et al. (2010) have reported that nifedipine, a calcium-channel blocker, blocks the advanced glycation end product (AGE)-induced tubular damage and also inhibits ICAM1 gene activity in tubular cells, which may have benefits in treatment of DN. Liu et al. (2010) have suggested that berberine can ameliorate renal dysfunction in diabetic rats by decreasing ICAM1 gene expression and nuclear factor-kappa B (NF-kappaB) activation. Taking together, the data from these studies suggest that ICAM1 may be a good candidate as target for drug development. Inhibition of ICAM1 gene activity may benefit in treatment of diabetes and DN.

## SUMMARY AND PERSPECTIVES

We have described genetic and biological studies of ICAM1 in diabetes and DN. Data implicate that ICAM1 can be used a biomarker for prediction of diabetes and DN and may also be serviced as a target for drug development. To reach these two objectives, however, we need to continuously put efforts into the study of ICAM1 in diabetes research. First, genetic polymorphisms in the *ICAM1* gene are associated with T1D, T2D and DN in T1D. But, no genetic association with DN in T2D has been reported. Although it is known that methylation modification of genomic DNAs and histone proteins is involved in the regulation of ICAM1 gene expression, no epigenetic study of this gene in diabetes and DN has been done. Second, clinical observations in diabetes patients and experimental studies with diabetic animal models have demonstrated that elevation of serum ICAM1 levels and overexpression of the gene in kidney tissues. Inhibition of ICAM1 gene activity with agents may improve the kidney function, suggesting that ICAM1 may be a target for drug development in treatment of diabetes and DN.

## Conflict of Interest Statement

The authors declare that the research was conducted in the absence of any commercial or financial relationships that could be construed as a potential conflict of interest.

## Abbreviations

ACR: urinary albumin/creatinine ratio
AER: albumin excretion rate
CD54: cluster of differentiation 54
DN: diabetic nephropathy
ESRD: end-stage renal disease
GFR: glomerular filtration rate
GoKinD: Genetics of Kidneys in Diabetes
HDL: high-density lipoprotein
HWE: Hardy– Weinberg equilibrium
ICAM1: intercellular adhesion molecule 1
LD: linkage disequilibrium
LDL: low-density lipoprotein
LFA: leukocyte adhesion protein
SNP: single-nucleotide polymorphism
T1DM: type 1 diabetes mellitus
T2DM: type 2 diabetes mellitus
UTR: un-translation region.
